# Non-metallic doped GeC monolayer: tuning electronic and photo–electrocatalysis for water splitting

**DOI:** 10.3389/fchem.2024.1425698

**Published:** 2024-10-01

**Authors:** Xiangxiang Ni

**Affiliations:** School of Automobile and Traffic Engineering, Guangzhou City University of Technology, Guangzhou, China

**Keywords:** single-layer carbon germanium, electronic, magnetism, doping, first principle

## Abstract

We conducted a first-principles study on the electronic, magnetic, and optical characteristics of non-metallic atoms (B, C, F, H, N, O, P, S, and Si) doped in single-layer carbon germanium (GeC). The findings indicate that the introduction of various non-metallic atoms into the monolayer GeC leads to modifications in its band structure properties. Different non-metallic atoms doped in single-layer GeC will produce both magnetic and non-magnetic properties. B-, H-, N-, and P-doped GeC systems exhibit magnetic properties, while C-, F-, O-, S-, and Si-doped single-layer GeC systems exhibit non-magnetic properties. Different non-metallic-doped single-layer GeC systems will produce semiconductor, semimetallic, and metallic properties. The C-, N-, O-, P-, S-, and Si-doped GeC systems still exhibit semiconductor properties. The H-doped GeC system exhibits semimetallic properties, while the B- and F-doped GeC systems exhibit metallic properties. Other than that, the doping of B, H, N, and P atoms can modulate the magnetism of single-layer GeC. Subsequently, we studied the influence of the doping behavior on the work function, where the work function of the single-layer GeC system doped with P atoms is very small, indicating that its corresponding doping system (P-doped GeC system) can produce a good field emission effect. In the optical spectrum, the doped systems have a certain influence in the far ultraviolet region. Furthermore, our results showed that S- and Si-doped single-layer GeC systems are conducive to photocatalysis compared to the single-layer GeC system.

## 1 Introduction

In recent years, it is well known that two dimensional (2D) materials (Lin et al., 2017; Bhimanapati et al., 2015; Chang et al., 2021; Khan et al., 2020; Dubertret et al., 2015), such as graphene (Geim, 2009; Grigorenko et al., 2012; Rao et al., 2009), have become exploration topics in fields including optoelectronics (Bonaccorso et al., 2010; Wu et al., 2023; Bao and Loh, 2012; Cui et al., 2021), field-effect transistors (Reddy et al., 2011), magnetism (Yazyev, 2010), electronics (Wu et al., 2013), and spin devices (Han et al., 2012). In addition, as a typical 2D material, single-layer carbon germanium (GeC) (Ma et al., 2021) can be discussed in many aspects, such as optics (Xu et al., 2016), electricity (Gao et al., 2019), and magnetism (Fan et al., 2021). According to the research, single-layer GeC has excellent performance in many fields and provides a solid theoretical foundation for future development.

The 2D form of germanene exhibits a folded structure, which is significantly different from the planar honeycomb structure of graphene. Despite these differences in configuration, both materials have the same dispersion relationship and form Dirac points (Soldano et al., 2010). Unlike graphene, GeC has a large band gap and possesses excellent optical and electrical properties. GeC has good potential for use in optoelectronic devices, solar cells, and other fields. The advancement of germanium material technology has led to the widespread application of semiconductor devices in various fields, including computers, television, mobile phones, and aerospace (Jin et al., 2015).

At the forefront of theoretical research, methods such as adsorption (Dąbrowski, 2001; Zhu et al., 2017; Zhu et al., 2016; Mantasha et al., 2020; Ren et al., 2022; Bagsican et al., 2017) and doping (Zhu et al., 2020; Feng et al., 2017; Zhang and Robinson, 2019; Wang et al., 2021), which are commonly known, are the most ideal way to study single-layer 2D materials. Luo et al. (2018) investigated the adsorption behavior of multiple transition metals (TMs) on phosphorene. The outcomes indicate that whole adsorption systems of transition metals exhibit high binding energies. Additionally, the study also examined the oxygen molecules on TM–phosphorene. The results indicate that all O_2_- (TM–phosphorene) systems, except for O_2_- (Pd–phosphorene), may lengthen the O-O bond and enable O_2_- (Co–phosphorene) to exhibit a half-metallic state. Chen et al. (2019) investigated a series of behaviors of original and TM-doped GaN-ML adsorbed by molecules such as H_2_S, NH_3_, and SO_2_. The results indicate that Fe and Mn atom doping can improve the adsorption capacity of certain molecules in their corresponding adsorption systems. In addition, the inter-reaction between the adsorbed molecules and the TM-doped GaN-ML is strong and, thus, can effectively cause converts in conductivity. Chegel (2023) found that when considering the doped structure, the band gap is influenced by the type of impurity present. Specifically, in a BC-doped [NC-doped] structure, a lower impurity concentration results in a smaller [larger] band gap. Hashmi and Hong (2015) proposed that transition metal-doped phosphorene layers can exhibit dilute magnetic semiconductor properties. They predicted that transition metal-doped phosphorene could serve as a potential next-generation material for spintronic applications. Ullah et al. (2015) used first-principle density functional theory (DFT) calculations to study the structure and electronic properties of beryllium (Be)-doped and Be and boron (B)-co-doped graphene systems. They discussed the relative stability of the doped systems and examined the effects of oxygen vacancies. The results indicate that oxygen vacancies tend to increase the lattice parameters and significantly reduce the bulk modulus. The defect formation energy is related to the computed crystal and covalent radii of the dopants, without showing a straightforward trend. Sun et al. (2016) investigated the geometry, electronic properties, and magnetism of transition metal (Sc-Ni)-doped germanene. Their analysis indicated that the magnetic moment originates from the localized d-orbitals of the transition metal atoms. Notably, in the case of Mn substitution at a concentration of 3.125%, a Curie temperature exceeding room temperature was achieved, highlighting the potential for magnetic storage applications. Inspired by these studies, we investigated some characteristics of single-layer GeC doped with non-metallic atoms.

The electronic, magnetic, and optical characteristics of non-metallic atoms doped in single-layer GeC are investigated by first principles. The next section introduces the methods, and the final section presents the research conclusions. Our research hopes to provide a theory for future nanodevices.

## 2 Calculation methods and models

All the research in the article is based on DFT from first principles. This article adopted a 4 × 4 × 1 supercell model of single-layer GeC, where C atoms are represented in brown, doped non-metallic atoms are represented in brown–yellow, and Ge atoms are represented in light purple. We considered two doping scenarios, namely, substitution of C atoms and Ge atoms. The VESTA program was utilized for the 3D visualization of volumetric data. The plane wave basis group was calculated using the projector augmented wave (PAW) method (Walter et al., 2008). The Perdew–Burke–Ernzerhof formula (Perdew et al., 1996) can deal with exchange-related effects between electrons. Van der Waals interactions were considered using the DFT-D3 approach (Slater and Kirkwood, 1931). The data were calculated by using DFT in the VASP package (Bekaroglu et al., 2010; Hafner, 2008; Liu and Ni, 2010). In the Brillouin zone, a 3 × 3 × 1 Monkhorst–Pack grid was used for k-point sampling. The mechanical convergence criterion was set to 0.01 eV Å^-1^. During the self-consistency, the convergence criterion for the system energy was set to 10–5 eV. The vacuum space was set to 20 Å.

When all doping systems have been optimized and a stable equilibrium state has been obtained, the formula of the doping energy (*E*_do_) is as follows:

$$E_{\text{do}}=E_{\text{Total}}\;–\;E_{\text{GeC}}\;–\;E_{\text{atom}}+E_{\text{Ge}/C},$$ (1)

where *E*_do_ represents the doping energy. $E_{\text{total}}$ , $E_{\text{GeC}}$ , and $E_{\text{atom}}$ are the overall energy of single-layer GeC doped with non-metallic atoms, the energy of single-layer GeC, and doped non-metallic atom energy, respectively. $E_{\text{Ge}/C}$ represents the energy of the substituted Ge or C atom. The charge transfer was calculated through Bader charges. The spin-polarized charge density of single-layer GeC doped with non-metallic atom systems was also calculated ( $\rho =\rho_{\text{spin}-\text{up}}-\rho_{\text{spin}-\text{down}}$ ).

Charge density difference (CDD) illustrates the transfer of charge between the non-metallic atoms and the single-layer GeC. The CDD for these doping systems was determined using the formula provided below:

$$\Delta \rho =\rho_{\text{Total}}-\rho_{\text{GeC}}-\rho_{\text{atom}},$$ (2)

where $\rho_{\text{Total}}$ , $\rho_{\text{GeC}}$ , and $\rho_{\text{atom}}$ denote the charge density of non-metallic atoms doped in the single-layer GeC, single-layer GeC, and the non-metallic atoms, respectively.

We also estimated the work function of non-metallic atoms doped in single-layer GeC:

$$w=E_{\text{Vac}}-E_{F},$$ (3)

where $E_{\text{Vac}}$ refers to the energy level of the vacuum, while $E_{F}$ represents the Fermi energy level.

## 3 Discoveries and discourses

**FIGURE 1:**
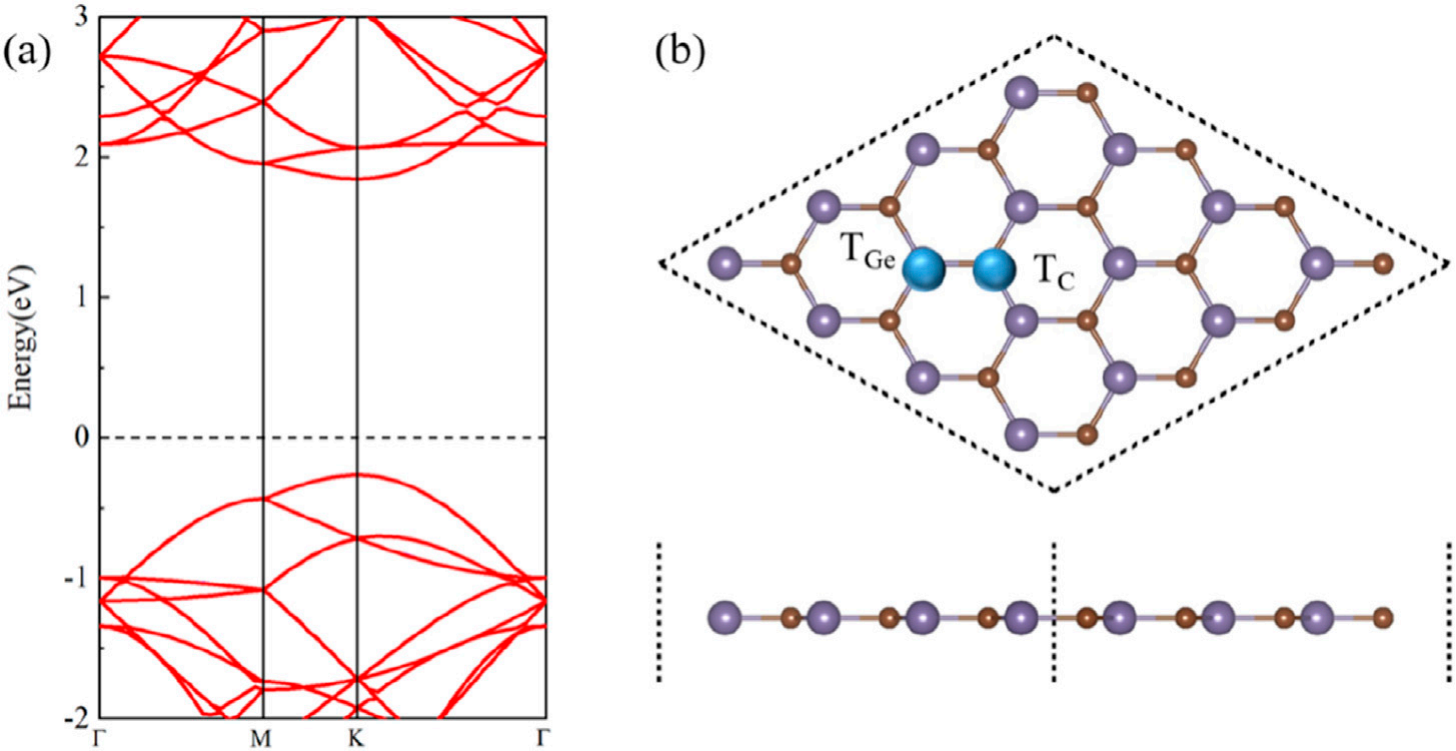
**(A)** Band structure and **(B)** crystal structure of single-layer GeC.

Figures 1A, B show the band structure and crystal structure of single-layer GeC, respectively, as shown in Figure 1. Figure 1A shows that the monolayer GeC exhibits characteristics of a direct band gap semiconductor. In Figure 1B, the blue ball represents the doping site. TGe is labeled as a non-metallic atom replacing the Ge atom, which is a site-doped Ge atom, while TC is labeled as a site-doped C atom. In addition, Table 1 also lists the doping energy (E_do_), magnetic moment (M_atom_), charge transfer (C), and work function (w) of non-metallic atom-doped single-layer GeC systems.

**TABLE 1:** Doping energy (*E*_do_), magnetic moment (*M*_atom_), charge transfer (*C*), and work function (*w*) of the non-metallic atom-doped single-layer GeC system.

Configuration	E_do_ (eV)	M_atom_ (μ_B_)	C (e)	w (eV)
B	−13.77	0.39	−1.805	4.97
C	−15.75	0.00	−0.151	4.65
F	−4.92	0.00	0.712	4.45
H	−4.82	3.00	−0.263	4.89
N	−14.00	0.92	0.830	3.73
O	−7.95	0.00	1.117	4.45
P	−13.17	1.00	−1.625	3.59
S	−10.42	0.00	−0.439	4.28
Si	−13.77	0.00	−2.476	4.69

**FIGURE 2:**
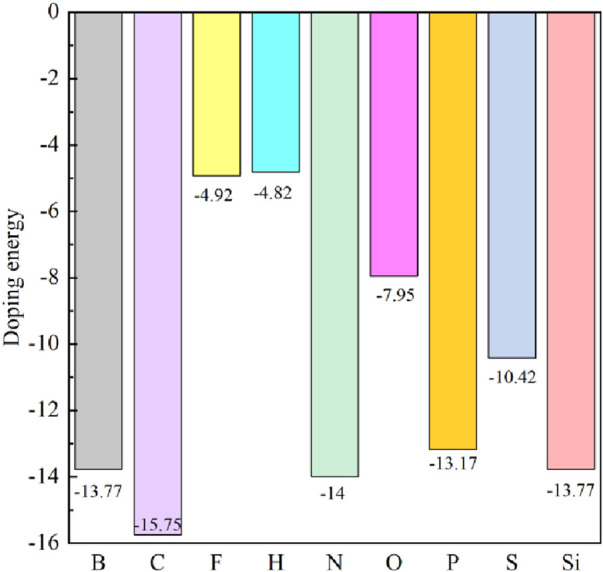
Doping energy of the single-layer GeC doped with non-metallic atoms.

Using the process of doping, we can discern the most stable doped systems. As shown in Figure 1, we first compared the doping energies between Ge atom and C atom doping, identifying the most stable doping structures at these two doping sites. Based on this comparison, we then determined the doping energies of nine different non-metallic atoms. The doping energy is calculated by Equation 1. Figure 2 shows the doping energies of these stable systems. Among the nine selected non-metallic atoms, it is noteworthy that the doping energy of the system doped with a C atom in the single-layer GeC exhibits the lowest doping energy, hence rendering it the most stable. Interestingly, our observations reveal that the doping energies for the systems doped with B and Si atoms are identical.

**FIGURE 3:**
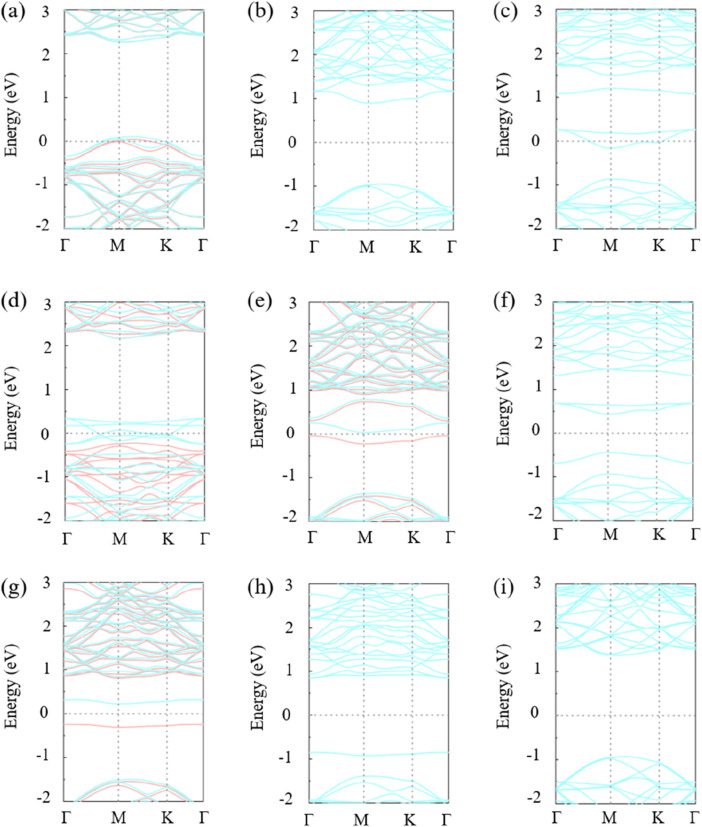
Band structures of single-layer GeC doped with non-metallic atoms: **(A)** B, **(B)** C, **(C)** F, **(D)** H, **(E)** N, **(F)** O, **(G)** P, **(H)** S, and **(I)** Si. The Fermi level is indicated by the light-gray dashed line, set to 0. The light-orange curve represents spin up, while the light-blue curve represents spin down.

The band structure of the monolayer GeC doped with non-metallic impurities is shown in Figure 3. The single-layer GeC system doped with B, H, N, and P exhibits magnetic properties, while the remaining five non-metallic atom-doped GeC systems do not produce magnetism. In other words, C-, F-, O-, S-, and Si-doped GeC systems exhibit non-magnetic properties. Figures 3A, C show that the spin-up and spin-down states intersect at the Fermi level, indicating that both figures depict metallic behavior. In other words, the B- and F-doped single-layer GeC system exhibits metallic properties. Therefore, the B-doped GeC systems exhibit magnetic metallic properties, and F-doped GeC systems exhibit non-magnetic metallic properties. Figure 3D shows that the spin-down state intersects at the Fermi level, so Figure 3D is semimetal. In other words, the H-doped single-layer GeC system exhibits semimetallic properties. Therefore, the H-doped system exhibits magnetic semimetallic properties. Six non-metallic atoms, i.e., C-, N-, O-, P-, S-, and Si-doped GeC, still exhibit semiconductor properties. The respective band gap values for C-, N-, O-, P-, S-, and Si-doped GeC systems are 1.843, 0.070, 0.872, 0.467, 1.696, and 2.295 eV.

**FIGURE 4:**
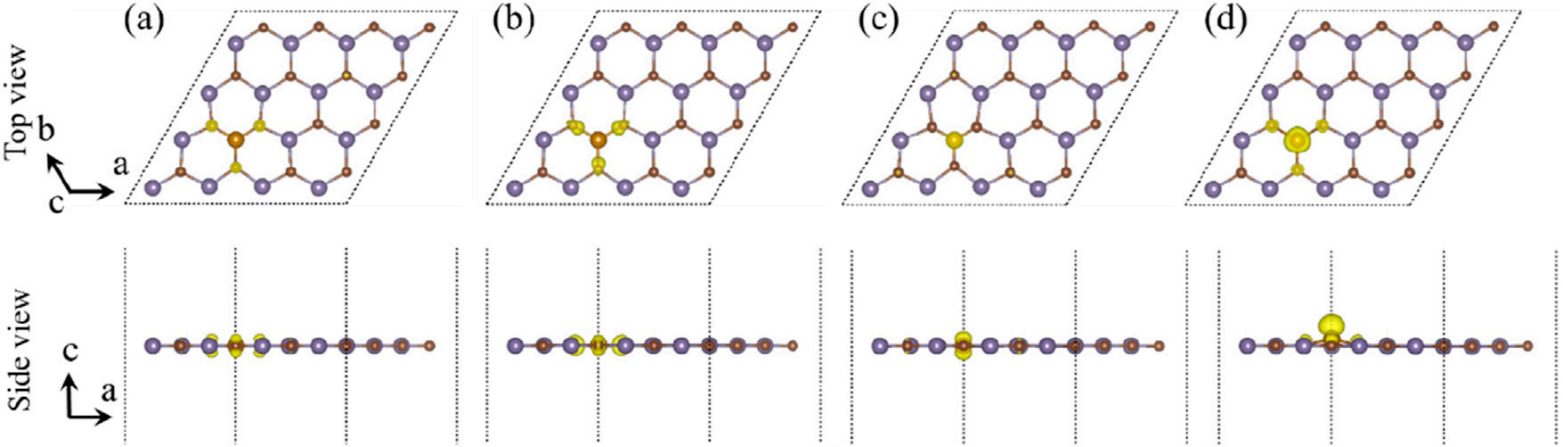
Spin-polarized charge densities of single-layer GeC doped with non-metallic atoms: **(A)** B, **(B)** H, **(C)** N, **(D)** and P. The yellow regions denote spin up, while the purple regions denote spin down.

As shown in Figure 4, in order to further investigate the properties of magnetism, the spin-polarized charge densities of B-, H-, N-, and P-doped single-layer GeC were studied. The corresponding magnetic moment data are given in Table 1. Upon computation, the magnetic moments of the B-, H-, N-, and P-doped GeC systems were determined as 0.39, 3.00, 0.92, and 1.00 μB, respectively. Consequently, it can be concluded that specific non-metallic atoms (B, H, N, and P atoms) possess the capability to modulate the magnetism of single-layer GeC. It is possible to apply it to magnetic equipment in the future.

**FIGURE 5:**
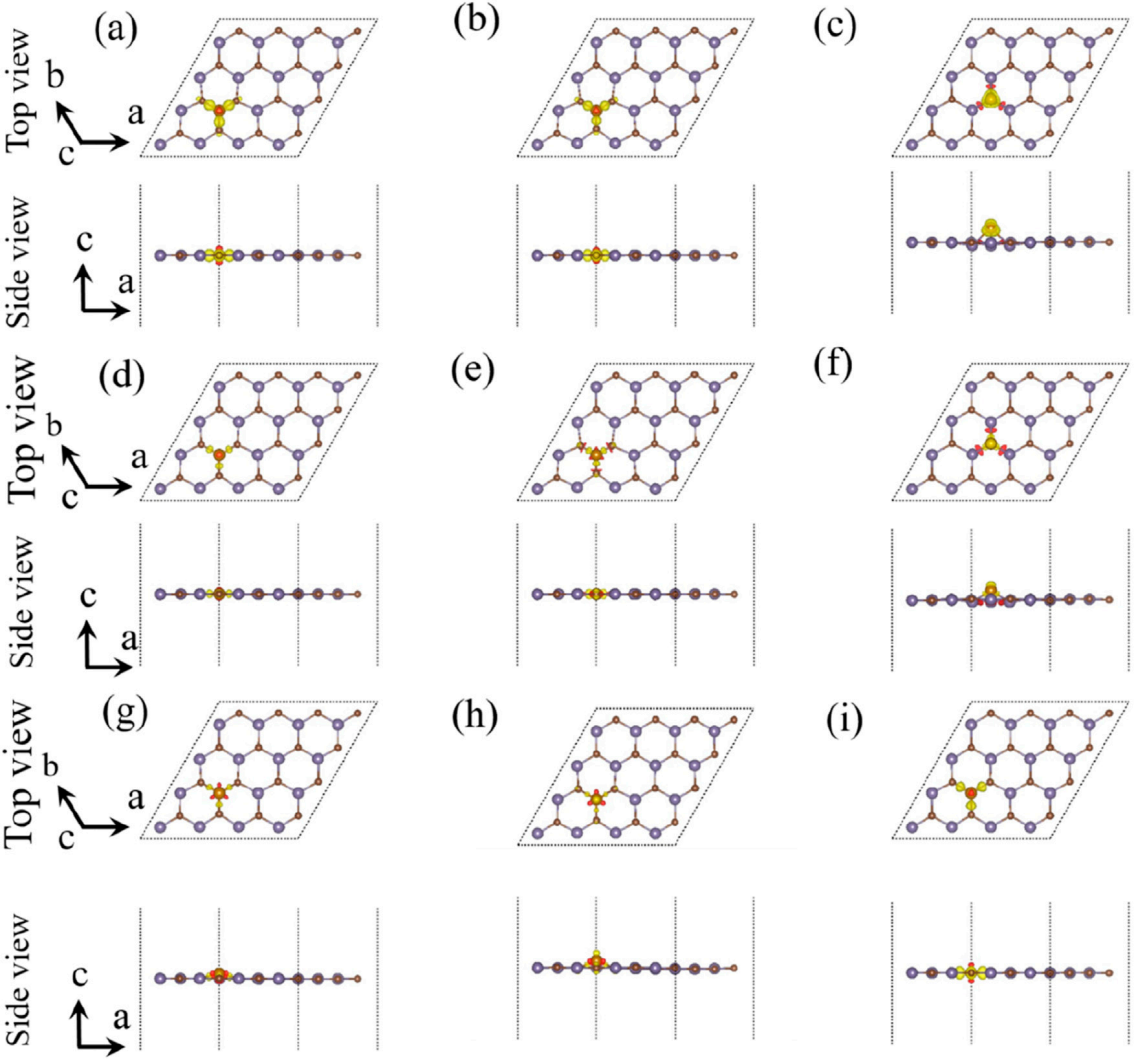
Charge density difference of the single-layer GeC doped with non-metallic atoms: **(A)** B, **(B)** C, **(C)** F, **(D)** H, **(E)** N, **(F)** O, **(G)** P, **(H)** S, and **(I)** Si. The yellow and red regions designate the accumulation of electrons and the depletion of electrons, respectively.

In order to further investigate the situation of electron transfer, we studied the charge density difference of the single-layer GeC doped with non-metallic atoms, as shown in Figure 5. After calculation by Equation 2, the amount of electron transfer was obtained. The relevant data are given in Table 1. After non-metallic atoms were doped in the single-layer GeC, the electrons lost by B, C, H, P, S, and Si atoms were 1.805 | e |, 0.151 | e |, 0.263 | e |, 1.625 | e |, 0.439 | e |, and 2.476 | e |, respectively. For the F, N, and O atom-doped GeC systems, they gained 0.712 | e |, 0.830 | e |, and 1.117 | e |, respectively. According to the data, it can be observed that the O atom gained the highest number of electrons, indicating that the charge transfers from the single-layer GeC to the corresponding O atom. In addition, B, C, H, P, S, and Si atoms lost electrons through doping the single-layer GeC, indicating that the charge has been transferred from B, C, H, P, S, and Si atoms to the single-layer GeC.

**FIGURE 6:**
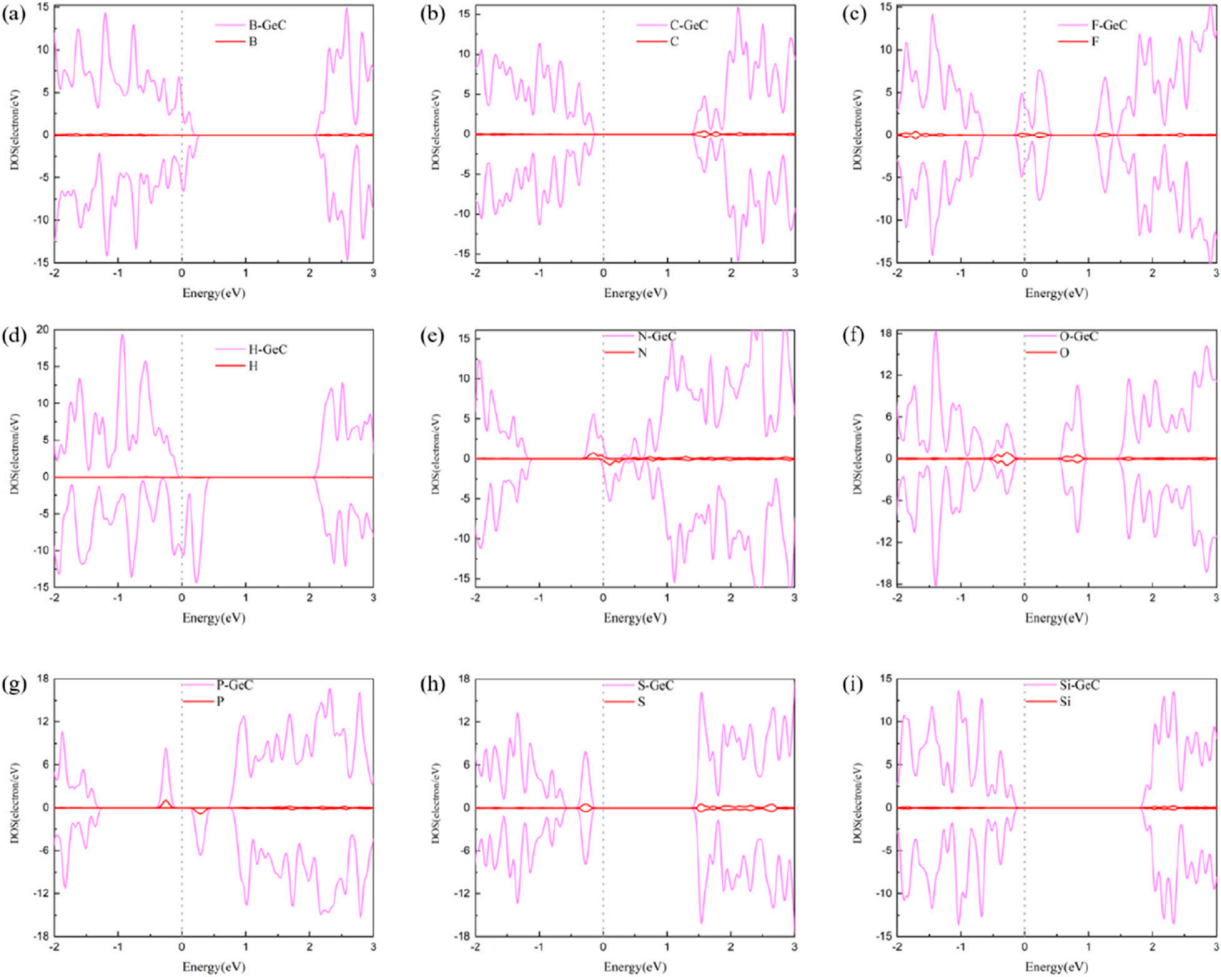
DOS of the single-layer GeC doped with non-metallic atoms: **(A)** B, **(B)** C, **(C)** F, **(D)** H, **(E)** N, **(F)** O, **(G)** P, **(H)** S, and **(I)** Si. The Fermi level is set to zero.

To delve deeper into the analysis of electron distribution of non-metallic atoms doped in the single-layer GeC systems, a density-of-states (DOS) plot was plotted, as shown in Figure 6. In Figures 6A, D, E, G, the DOSs of the spin-up and spin-down orbitals are mostly asymmetric, suggesting that their corresponding systems for the B-, H-, N-, and P-doped single-layer GeC systems exhibit magnetism. Conversely, in Figure 6, the DOSs of the spin-up and spin-down orbitals in Figures 6B, C, F, H, I are mostly symmetric, indicating that their corresponding systems for the C-, F-, O-, S-, and Si-doped single-layer GeC systems exhibit non-magnetic properties. In addition, Figures 6A–I show that the spin-up and spin-down orbitals were predominantly contributed by the single-layer GeC.

**FIGURE 7:**
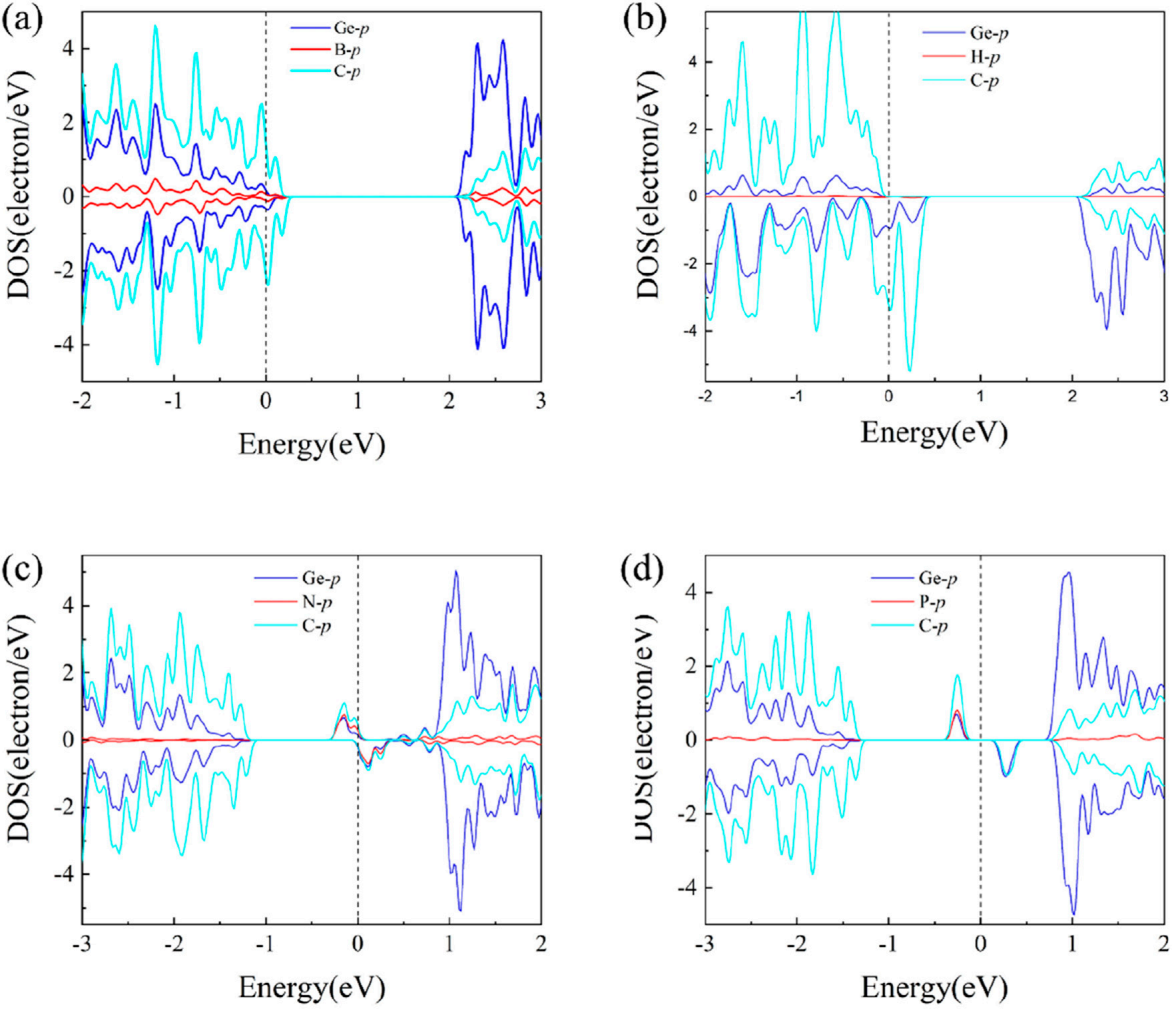
Atomic orbital diagrams of the **(A)** B-, **(B)** H-, **(C)** N-, and **(D)** P-atom-doped single-layer GeC systems.

In order to further explore the mechanisms of magnetism induced in the B-, H-, N-, and P-doped GeC monolayers, we plotted and analyzed the atomic orbital diagrams of these doping systems in Figure 7. For the four non-metallic atoms doped in the single-layer GeC systems, the orbital diagrams reveal notable shifts in the spin-up and spin-down states in either the Conduction band minimum (CBM) or Valance band maximum (VBM) regions. Clearly, this density of electronic states is attributed to the B-*p*, H-*p*, N-*p*, and P-*p* states in the CBM or VBM regions. The asymmetry between spin-up and spin-down states of B-*p*, H-*p*, N-*p*, and P-*p* induces polarization of the spin states of C-*p* electrons near the Fermi level. This results in a shift in the density of states around the Fermi level, which is consistent with the magnetic properties observed in the band structure. Thus, magnetism primarily originates from the polarization of the surrounding C-*p* spin states induced by the *p-*electron orbitals of the non-metal elements (B, H, N, and P), leading to the generation of a net magnetic moment in the system.

**FIGURE 8:**
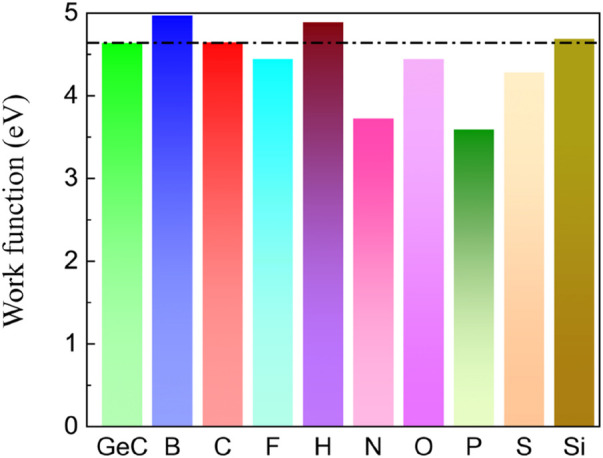
Work functions of the single-layer GeC and single-layer GeC doped with non-metallic atoms.

The examination of the disparity in charge density, as shown in Figure 5, reveals the phenomenon of charge transfer resulting from the introduction of non-metallic atoms doped in the single-layer GeC. The electron transfer can cause a rearrangement of charges, altering the work function. Therefore, we investigated the changes in the work function of monolayer GeC doped with non-metallic atoms. The work function is calculated by Equation 3. Figure 8 shows that the work function value of monolayer GeC is measured at 4.64 eV. We can conclude that after doping with B, C, H, and Si atoms, the work function of single-layer GeC increases. The corresponding work function value of the B-doped GeC system is 4.97 eV, that of the corresponding C-doped GeC system is 4.65 eV, that of the corresponding H-doped GeC system is 4.89 eV, and that of the related Si-doped GeC system is 4.69 eV. However, conversely, the work functions decrease after single-layer GeC is doped with F, N, O, P, and S atoms. The corresponding work functions for the systems (F-, N-, O-, P-, and S-doped GeC systems) are 4.45, 3.73, 4.45, 3.59, and 4.28 eV, respectively. Furthermore, we can conclude that the work function of single-layer GeC doped with P atoms is the lowest, indicating that its corresponding system (P-doped GeC system) can have a better field emission effect.

**FIGURE 9:**
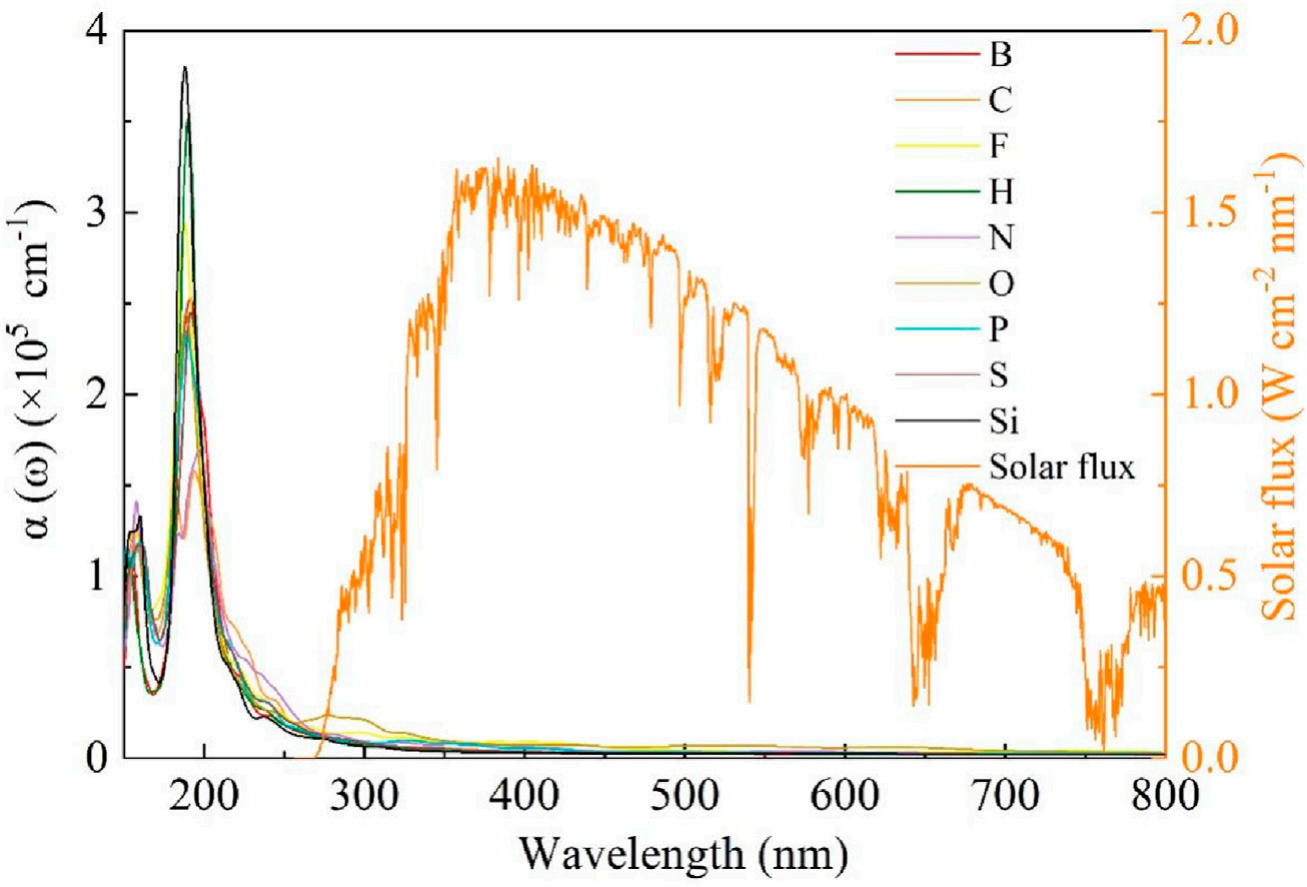
Doping spectrum of single-layer GeC doped with non-metallic atoms.

The doping spectra of non-metallic atom-doped single-layer GeC are shown in Figure 9. We examined the doping spectra within the wavelength range of 150–800 nm. According to the investigation, non-metallic atoms F, H, and Si exhibit high peaks between 150 and 200-nm wavelengths. In Si-, H-, and F-doped single-layer GeC systems, strong peaks appear in descending order, corresponding to wavelengths of 188, 190, and 188 nm, respectively. Furthermore, Figure 8 shows that the spectra of the other bands are relatively flat. After 300 nm, the spectra of all non-metallic atom-doped single-layer GeC systems tend to flatten out. This means that there is almost no prominent effect in the visible light ranges. Consequently, it can be concluded that the single-layer GeC doped with non-metallic atoms exhibits more pronounced characteristics in the far ultraviolet band (wavelengths <200 nm), while a doping effect is particularly weak in the near-infrared and visible-light ranges.

**FIGURE 10:**
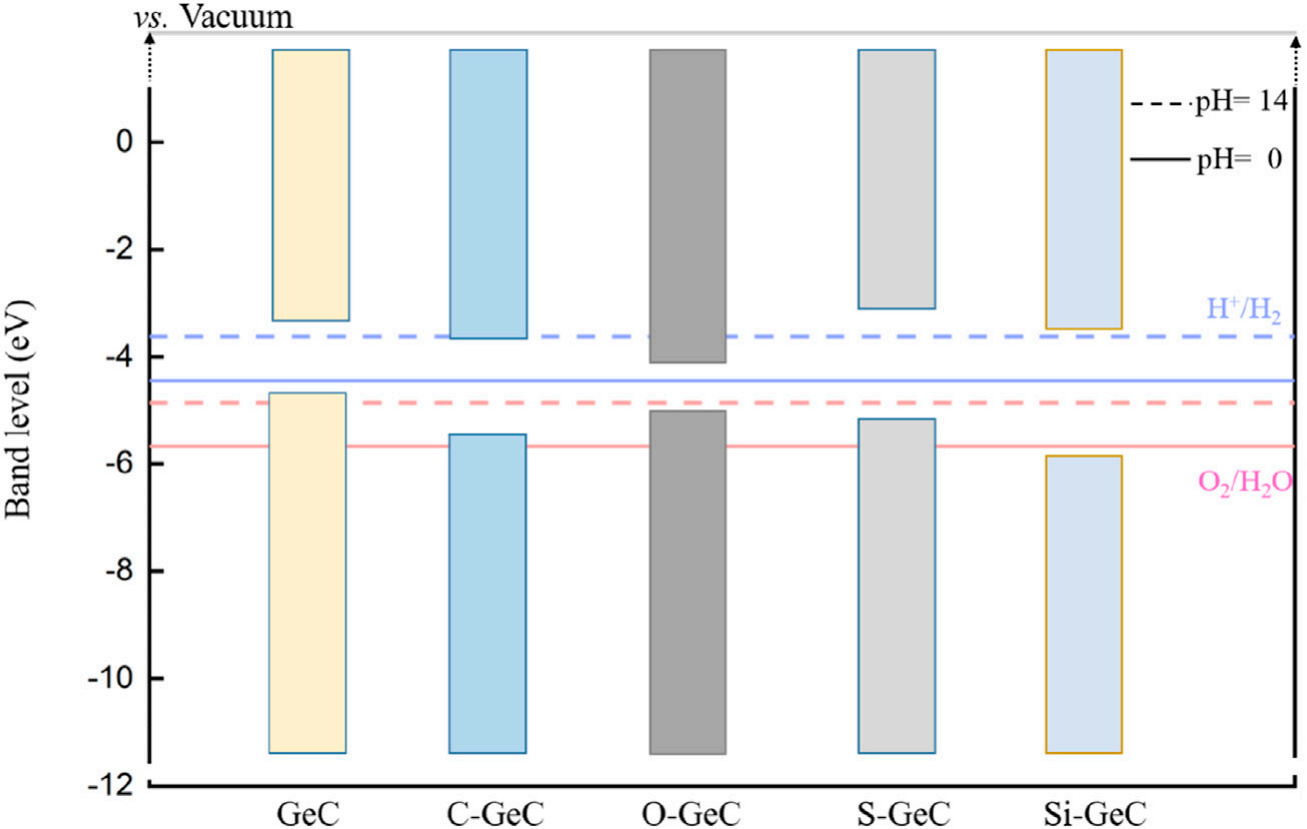
Band-edge positions of the doping system. The potential at pH = 14 is represented by the dotted line, while the potential at pH = 0 is indicated by the solid line.

Figure 10 shows the band-edge location of the doping system. The potential at pH = 14 is represented by the dotted line, whereas the potential at pH = 0 is indicated by the solid line. Both the C- and O-doped single layer GeC system does not facilitate redox reactions. In other words, these three doping systems are incapable of generating either hydrogen or oxygen. However, it is worth noting that the S-doped single-layer GeC system is capable of undergoing redox reactions at pH = 14, meaning that it is capable of generating both hydrogen and oxygen under pH = 14. Additionally, the Si-doped single-layer GeC system can undergo redox reactions under both pH = 0 and pH = 14 conditions. In other words, it is capable of generating both hydrogen and oxygen under these conditions. Our results show that S- and Si-doped single-layer GeC systems are conducive to photocatalysis compared to the single-layer GeC system.

**FIGURE 11:**
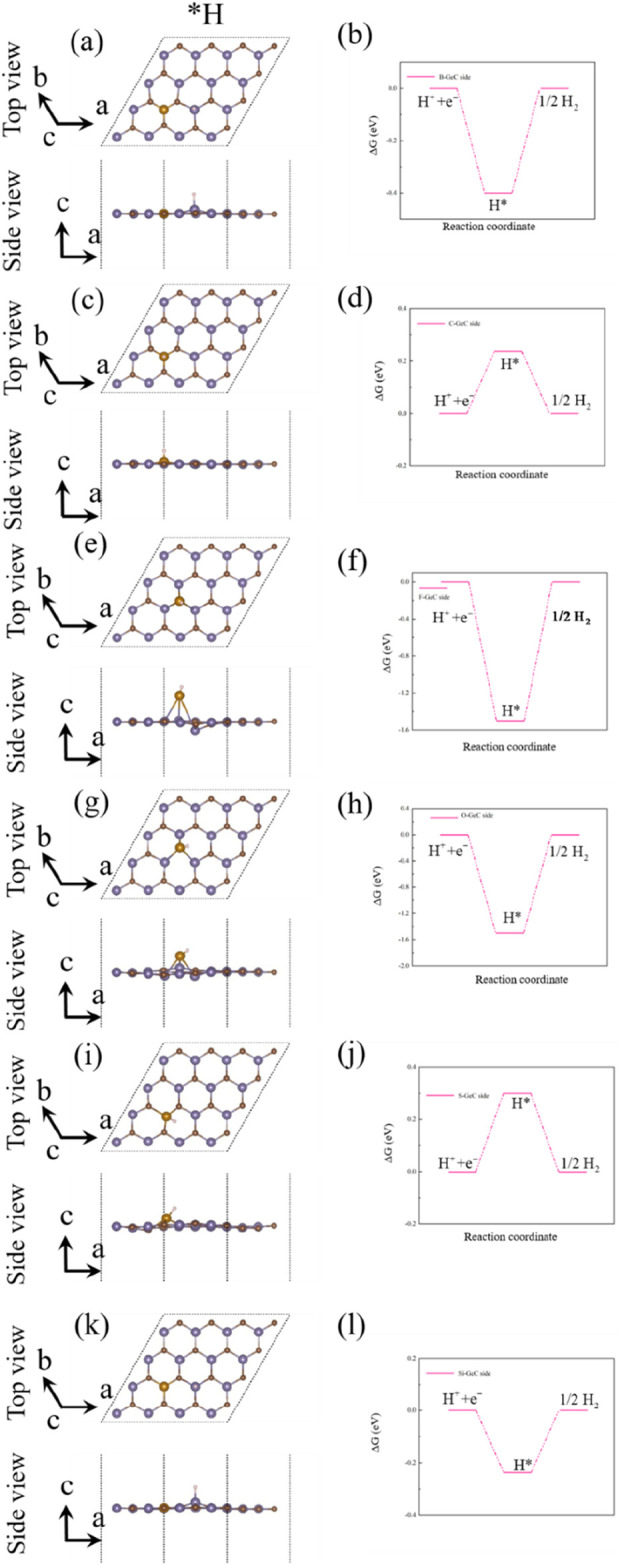
**(A)**, **(C)**, **(E)**, **(G)**, **(I)**, and **(K)**. Most stable adsorption site and crystal structure of the hydrogen adsorption state (*H) in the HER. **(B)**, **(D)**, **(F)**, **(H) (J)**, and **(L)** GFE curves of the HER of the non-metallic atom-doped single-layer GeC, when U = 0 V.

Figures 11A, C, E, G, I, K show the most stable adsorption site and crystal structure of the hydrogen atom adsorption state (*H) in the hydrogen evolution reaction (HER). Above is the top view, and below is the side view. Figures 11B, D, F, H, J, L show the Gibbs free energy (GFE) curve of the HER for the single-layer GeC doped with non-metallic atoms. For the non-metallic B-atom-doped single-layer GeC system, the ΔG*H value is 0.4 eV (U = 0 V), corresponding to an overpotential (ηHER) of 0.4 V for the HER. In the case of the non-metallic C-atom-doped single-layer GeC system, ΔG*H is 0.002 eV (U = 0 V), suggesting an overpotential (ηHER) of 0.002 V for the HER. The non-metallic F-atom-doped single-layer GeC system exhibits a ΔG*H value of 1.5 eV (U = 0 V), leading to an overpotential (ηHER) of 1.5 V for the HER. Similarly, the non-metallic O-atom-doped and S-atom-doped single-layer GeC systems display ΔG*H values of 1.5 eV (U = 0 V) and 0.3 eV (U = 0 V), respectively, resulting in overpotentials (ηHER) of 1.5 and 0.3 V for the HER, respectively. In the case of the non-metallic Si-atom-doped single-layer GeC system, ΔG*H is 0.3 eV (U = 0 V), suggesting an overpotential (ηHER) of 0.3 V for the HER. Among these six doping systems, the F-atom-doped GeC system exhibits the most pronounced GFE curve for the HER.

## 4 Conclusion

Based on the first principles, the electronic, magnetic, and optical behaviors of non-metallic atom (B, C, F, H, N, O, P, S, and Si)-doped single-layer GeC were investigated to explore the characteristics of doping systems. Through a series of studies, it was found that the non-metallic doping behavior in single-layer GeC can modulate its performance. After doping, B-, H-, N-, and P-doped GeC systems exhibit magnetic properties, while C-, F-, O-, S-, and Si-doped GeC systems exhibit non-magnetic properties. The B-doped GeC system exhibits magnetic metallic properties, the F-doped GeC system exhibits non-magnetic metallic properties, and the H-doped GeC system displays magnetic semimetallic properties. This implies that non-metallic atom doping can adjust the band structure of monolayer GeC. The work function of non-metallic atom-doped GeC systems also yields different results. Some doping systems have work functions larger than the intrinsic value, while others have work functions smaller than the intrinsic value. The work functions of B-, C-, F-, N-, O-, P-, S-, and Si-doped GeC systems are 4.97, 4.65, 4.89, 4.45, 3.73, 4.45, 3.59, 4.28, and 4.69 eV, respectively. Specifically, the work function of the single-layer GeC system doped with P atoms is remarkably small. Therefore, it is expected to have certain advantages in the realm of efficient field emission. In the doping spectrum, the presence of non-metallic atoms in single-layer GeC is more prominent in the far ultraviolet region (wavelengths <200 nm). Furthermore, the band-edge position of the doped system shows that the doped system (S- and Si-doped single-layer GeC system) can undergo redox reactions. Through this research, we aim for further developments in the field of optoelectronics and photo–electrocatalysis.

## Data Availability

The original contributions presented in the study are included in the article/Supplementary Material; further inquiries can be directed to the corresponding author.
